# Seropositivity to Herpes Simplex Virus Antibodies and Risk of Alzheimer's Disease: A Population-Based Cohort Study

**DOI:** 10.1371/journal.pone.0003637

**Published:** 2008-11-04

**Authors:** Luc Letenneur, Karine Pérès, Hervé Fleury, Isabelle Garrigue, Pascale Barberger-Gateau, Catherine Helmer, Jean-Marc Orgogozo, Serge Gauthier, Jean-François Dartigues

**Affiliations:** 1 INSERM, U897, Bordeaux, France; 2 Universite Victor Segalen Bordeaux 2, Bordeaux, France; 3 Universite Victor Segalen Bordeaux 2, Laboratoire de Virologie, Bordeaux, France; 4 McGill University, Centre for Studies in Aging, Montreal, Quebec, Canada; Cincinnati Childrens Hospital, United States of America

## Abstract

**Background:**

Herpes Simplex Virus (HSV) infection has been proposed as a possible risk factor of Alzheimer's Disease (AD) notably because it is neurotropic, ubiquitous in the general population and able to establish lifelong latency in the host. The fact that HSV was present in elderly subjects with AD suggests that the virus could be a co-factor of the disease. We investigated the risk of developing AD in anti-HSV immunoglobulin G (IgG) positive subjects (indicator of a lifelong infection to HSV) and IgM-positive subjects (indicator of primary infection or reactivation of the virus) in a longitudinal population-based cohort of elderly subjects living in the community.

**Methods:**

Cox proportional hazard models were used to study the risk of developing AD according to the presence or not of anti-HSV IgG and IgM antibodies, assessed in the sera of 512 elderly initially free of dementia followed for 14 years.

**Results:**

During the follow-up, 77 incident AD cases were diagnosed. Controlled for age, gender, educational level and Apolipoprotein E4 (APOE4) status, IgM-positive subjects showed a significant higher risk of developing AD (HR = 2.55; 95% CI [1.38–4.72]), although no significant increased risk was observed in IgG-positive subjects (HR = 1.67; 95%CI [0.75–3.73]). No modification effect with APOE4 status was found.

**Conclusion:**

Reactivation of HSV seropositivity is highly correlated with incident AD. HSV chronic infection may therefore be contributive to the progressive brain damage characteristic of AD.

## Introduction

In the general population, Herpes Simplex Virus (HSV) is highly prevalent (more than 70% after age 50) [1]–[3]. This virus persists latently in the peripheral nervous system, and periodically reactivates with production of active virus. Herpes Simplex Encephalopathy (HSE) is a rare but very severe acute infection of the central nervous system [4]. Although it has a very different course from Alzheimer's disease (AD), it leads to the occurrence of bilateral hippocampal-inner temporal lesions resulting in profound verbal memory loss, characteristic of AD. On the basis of this hippocampal and temporal tropism of the virus, HSV was proposed as a candidate environmental risk factor for AD [5]. Some studies found that HSV has been detected in the brain of many AD patients, both by direct detection of virus DNA by polymerase chain reaction (PCR) [6] and by the detection of intrathecal antibodies [7]. However, as the virus was also frequently detected in normal brains of aged individuals, it is unlikely that HSV infection is the only cause of the disease, but it may participate in the pathogenic process.

The fact that the frequency of HSV DNA-positive subjects was not different between AD and control subjects [6] and that intrathecal IgG antibodies were detected in a similar proportion of patients with AD and elderly controls [7] indicates that chronic HSV infection alone is not univocally associated with AD. It has been suggested, however, that the risk of developing AD in subjects positive for HSV DNA presence in the brain who carried apolipoprotein E ε4 allele (APOE-ε4) was several fold that of individuals possessing only one or neither of these factors [8]. However, this finding remains controversial as it has not been confirmed by another study [9].

Few studies have investigated the seroprevalence of anti-HSV antibodies in AD. Renvoize et al [10] found no statistically significant difference in serum antibody titres to HSV in a sample of 33 AD patients and 28 controls. Ounanian et al [11] in a sample of 19 AD patients and 21 controls, showed increased titres of antibodies to HSV in the control group but the proportion of HSV-positive subjects was not different between AD and control groups. These studies were performed on small samples of individuals and with IgG antibodies only, which characterise past infections. IgM antibodies, which characterise primary infections or reactivations, have not been investigated. Our objective is to assess the risk of developing AD according to the baseline anti-HSV-1 or HSV-2 immunoglobulin status (IgG and IgM) over a 14-year period of follow-up in a large prospective population-based study of elderly subjects.

## Methods

This study was part of the PAQUID (Personnes Agees QUID) research programme, a prospective cohort study of normal and pathological cerebral aging, composed of a randomly selected sample of non-institutionalised individuals aged 65 years and over living in the south west of France. The methodology of this study has already been extensively described [12]. The survey started in 1988, following 3777 subjects who were interviewed at home by trained psychologists one, three, five, eight, 10, 13 and 15 years after the baseline visit. Subjects who agreed to participate in the study gave their written informed consent; the study was approved by the Ethics Committee of the University Hospital of Bordeaux. At each visit, cognitive performances were evaluated using a comprehensive battery of neuropsychological tests including the Mini Mental State Examination (MMSE) [13]. After the psychometric evaluation, the psychologists systematically completed an evaluation of the DSMIII-R criteria for dementia [14] and subjects who met these criteria were subsequently seen by a senior neurologist who confirmed the diagnosis of dementia. Alzheimer's disease was diagnosed according to the National Institute of Neurological and Communicative Disorders and Stroke/the Alzheimer's Disease and Related Disorders Association (NINCDS/ADRDA) criteria [15].

At the first year visit in 1989, a subsample of 591 volunteers agreed to have a blood sample. Serum samples were frozen and stored in liquid nitrogen for subsequent analysis. The samples were analysed in 2007 to assess seropositivity to HSV. The detection of IgM and IgG antibodies to HSV was performed following the manufacturer's recommendations (Enzygnost Anti HSV/IgM and IgG, Dade Behring, Marburg, Germany) from 10 µl of serum for each parameter. The Enzygnost Anti-HSV/IgG (and Anti-HSV/IgM) is an ELISA for the quantitative determination of human IgG (respectively IgM) antibodies to HSV in serum. Subjects were considered positive for an optical density greater or equal to 0.1. IgG titres were expressed in international units per millilitre (IU/ml). Twenty eight subjects already demented at the time of blood collection were not included in the present analysis; in addition, 51 subjects who died or refused to participate to the follow-up after the blood sampling were also excluded. A total of 512 were then included in the present study. Subjects had only one blood sampling and no lumbar puncture was performed during the follow-up.

Statistical analysis: Cumulative incidence rates of AD were estimated. The cumulative incidence rates which measure the proportion of individuals who develop the disease during a specified period of time were obtained by the Kaplan-Meier method. The hazard ratios (HR) of AD and 95% confidence intervals (95% CI) were estimated using a Cox proportional hazard model. The association between IgG and IgM status and the onset of AD was controlled for age at inclusion, gender, educational level (primary school with diploma vs no schooling or primary school without diploma) and APOE-ε4 allele. To further investigate the evolution of the cognitive performance over time, the annual rate of change in MMSE score was modelled by a linear mixed model [16]. The adjusted model included terms for IgG or IgM status, age at baseline, gender, education level, APOE-ε4, time (time in years since the baseline visit, i.e. the blood sampling) and interaction terms between time and each covariate. The parameters for time interactions represent the estimated effect of the covariate on the annual rate of change since baseline. As the distribution of MMSE scores was not normal, we analyzed the square root of the number of errors, as proposed by Jacqmin-Gadda et al [17]. Indeed, these authors stated that, after transformation, graphical examination of residuals indicated that the hypotheses of normality and homoscedasticity were acceptable. Therefore, we chose to apply the same transformation to our data. All statistical analyses were done using SAS, version 9.1 (SAS institute, Inc., Cary, North Carolina).

## Results

Among the 512 non-demented subjects at inclusion, 99 developed a dementia (including 77 AD) over 8.2 years (SD = 4.4) of follow-up on average. The main characteristics of the subjects are given in table 1. As expected, subjects with incident dementia were older at inclusion. Incident AD cases were more frequently women and APOE-ε4 bearers. At baseline, 424 subjects (82.8%) were IgG-positive and 43 (8.4%) were IgM-positive. Only 86 (16.8%) subjects were negative for both IgG and IgM. Among the 43 IgM-positive subjects, only 2 were IgG-negative. Therefore, it is likely that most of the IgM-positive subjects showed recent reactivation of the virus. Unfortunately, no information was available about clinical manifestations of the infection to confirm this hypothesis.

**Table 1:** Main characteristics of the sample according to dementia status.

	Non demented	Incident Alzheimer's disease	Other incident dementia
Number of subjects	413	77	22
Age at inclusion. mean (sd)	72.8 (5.8)	75.8 (6.0)	74.4 (5.7)
Age at diagnosis or censorship. mean (sd)	82.8 (5.7)	85.8 (6.0)	85.4 (6.0)
Female gender (%)	52.5	71.4	59.1
High educational level (%)	76.5	61.0	77.3
APOE ε4 allele (%)	20.7	29.3	31.8
Anti-HSV IgG positive (%)	81.1	89.6	90.9
Anti-HSV IgM positive (%)	7.0	16.9	4.5

PAQUID study, n = 512.

**Figure 1:**
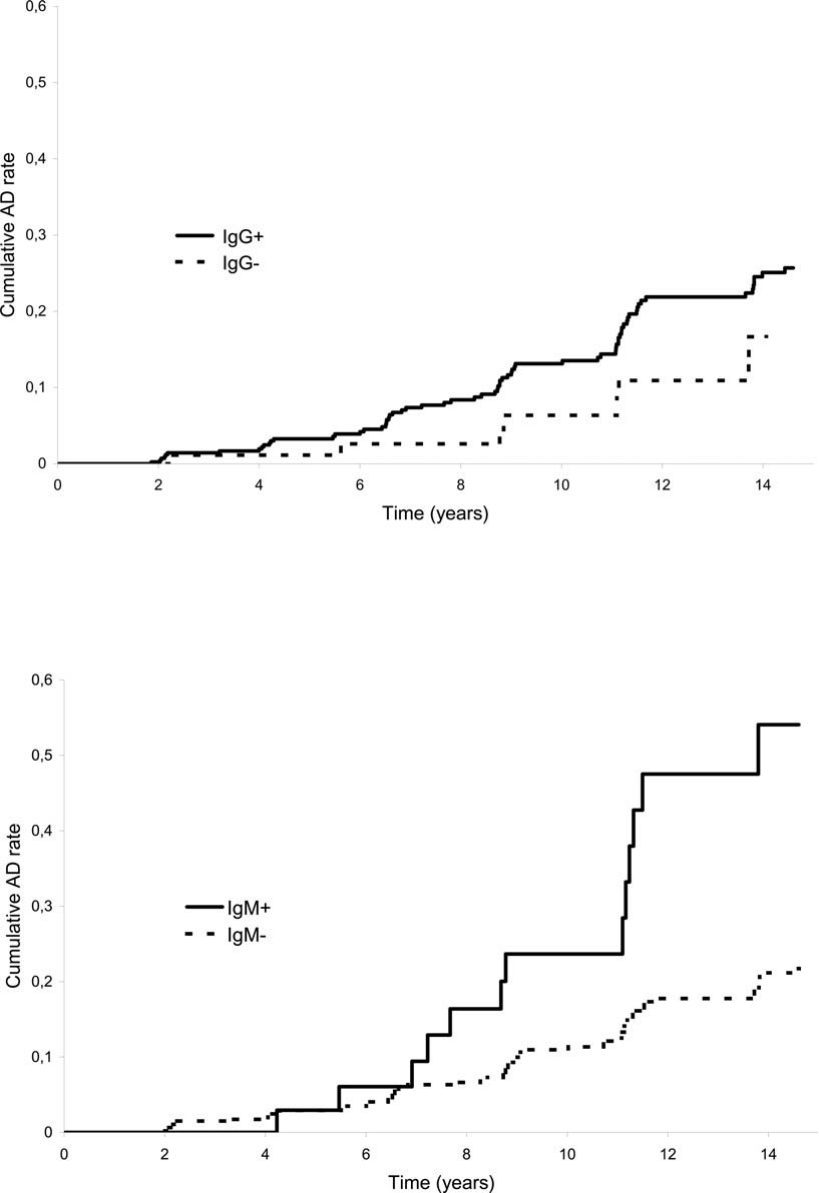
Cumulative Alzheimer's disease rate according to anti-HSV IgG (top) or anti-HSV IgM (bottom) status. Kaplan-Meier estimates, PAQUID study, n = 512.

During the follow-up, 69 (16.3%) of the 424 IgG-positive subjects, and 8 (9.1%) of the 88 IgG negative subjects developed AD. Among IgG-positive subjects, mean IgG titres were not significantly different between non-demented and demented subjects (mean (sd) :141 (57.3) and 151 (58.0), respectively, p = 0.13 ; median : 143 and 151 IU/ml, respectively). Thirteen (30.2%) of the 43 IgM-positive and 64 (13.7%) of the 469 IgM-negative subjects developed AD. The cumulative AD rate curves according to IgG and IgM status are displayed in figure 1. HSV-positive subjects showed a greater cumulative probability of developing AD than HSV-negative ones for both IgG and IgM. Cumulative AD rates were about 25% (95% CI: 0.08–0.39) in IgM positive subjects after 10 years of follow-up and reached a frequency of more than 50% (95% CI: 0.33–0.76) after 14 years.

After controlling for age, gender, educational level, APOE-ε4 and baseline MMSE, the hazard ratio of developing AD was not significantly greater in IgG-positive subjects (Table 2). In contrast, IgM-positive status at baseline was strongly associated with an increased risk of developing AD (Table 3). No modification effect was found between APOE and Ig status: p = 0.49 for IgM and p = 0.94 for IgG.

**Table 2:** Hazard ratio of developing AD according to Anti-HSV IgG status.

	HR	95% CI	p-value
Anti-HSV IgG status	1.67	0.75–3.73	0.21
Age	1.12	1.07–1.17	0.0001
Female gender	1.51	0.88–2.60	0.13
High educational level	0.92	0.53–1.60	0.77
APOE-ε4 allele	2.32	1.35–3.99	0.002
MMS score	0.83	0.75–0.92	0.0003

Cox Model, PAQUID study, n = 512.

**Table 3:** Hazard ratio of developing AD according to Anti-HSV IgM status.

	HR	95% CI	p-value
Anti-HSV IgM status	2.55	1.38–4.72	0.003
Age	1.12	1.07–1.17	0.0001
Female gender	1.48	0.86–2.56	0.16
High educational level	0.87	0.49–1.52	0.62
APOE-ε4 allele	2.00	1.18–3.41	0.011
MMS score	0.82	0.74–0.91	0.0002

Cox Model, PAQUID study, n = 512.

No significant association was found when other types of dementia (n = 22) were compared to non-demented subjects (n = 413): for IgG-positive subjects, HR = 2.16, (95% CI : [0.48 ; 9.69] p = 0.31) and HR = 0.61, (95% CI : [0.08 ; 4.68], p = 0.63) for IgM-positive subjects.

A linear mixed model was fitted to analyze the evolution of the square root of the number of errors to the MMSE over a 14-year period. After adjustment for age, sex, education level and APOE-e4, IgG status was marginally associated with the baseline score (β = 0.14, p = 0.056). The parameter was greater than 0 and this indicated that the number of errors at baseline tended to be higher for IgG positive subjects. The time parameter (β = 0.017, p = 0.17) showed a negligible and non significant increase of the number of errors over time. At baseline, IgM positive subjects tended to make fewer errors (β = −0.069, p = 0.50) than IgM negative ones, but the number of errors increased significantly over time (β = 0.035, p = 0.041).

## Discussion

The present cohort is the first to show the increased risk of AD in elderly subjects with a positive detection of anti-HSV IgM antibodies in a large population-based prospective study, after adjustment for known risk factors of AD, i.e. age, education and APOE status. This higher risk of dementia seems specific of AD, since the HR for other dementias was not significant, but the corresponding sample was small. In contrast, subjects with positive detection of anti-HSV IgG antibodies in the sera were not at higher risk of AD than IgG-negative subjects. This last result is in accordance with previous case-control clinical series [10], [11]. These results were confirmed when analysing the evolution of cognitive performance measured by the MMSE. This analysis did not rely only on a small sample of demented subjects and confirmed the greater cognitive decline of IgM positive subjects. Strandberg et al [18] analysed cognitive decline in 383 elderly cardiovascular subjects according to viral burden (3 herpesviradae : HSV1, HSV2, Cytomegalovirus (CMV)) and bacterial burden (*C pneumoniae*, *M Pneumoniae*) and showed a greater cognitive decline with viral burden in a stepwise manner. The HR of developing cognitive decline was 1.8 in subjects with 2 seropositivities and 2.3 in subjects with 3 seropositivities compared to subjects with 0 or 1 seropositivity. Aiello et al [19] showed a higher rate of cognitive decline over a 4 year period in subjects with the highest CMV antibody levels and no association between cognitive decline and HSV antibody levels. In this study, HSV-1 IgG antibodies were assessed and, as in the other studies examining IgG antibodies, it is likely that the titre remained unaltered between and during periods of recrudescence.

In this study, the sub-type of HSV virus (HSV-1 or HSV-2) was not determined. Since HSV-1 is more prevalent than HSV-2 [3], it is most likely that subjects were infected with HSV-1 virus. In addition, HSE caused by HSV-2 is very rare in adults. Further research is needed to analyse the impact of each virus type on the risk of AD.

Contrary to the findings of Itzhaki et al [8] in the comparison of brains of AD patients and controls, we did not find any interaction between APOE status and IgM-positive status on risk of AD. These negative results could be related to a lack of statistical power of our study since only 12 subjects were APOE4 bearers and IgM-positive. In our sample, we did not diagnose any case of herpes encephalitis that involves acute infection of the brain [20] whereas AD shows a progressive cognitive evolution.

Since IgM antibodies are present in the blood for a limited time period (the IgM response tends to decline within about one to two months [21], but in HSE, IgM persisted at a range from 56 days to 328 days [22]), IgM positive status indicated that HSV reactivation was recent. In addition, a high prevalence of positive IgM results among patients with established HSV infection [23] has been observed. As antibodies detection was performed only once in our study, we do not have any information of possible later reactivation of the virus. However, we may hypothesise that we had identified subjects involved in a chronic infection to regularly reactivating HSV, as that was well known for other HSV manifestations like cold sores. As no clinical information was collected about the occurrence of cold sores, we do not have any data to support this hypothesis.

Several interpretations could explain these findings. HSV1, which causes the rare and very severe herpes encephalitis, could possibly also produce a milder and chronic brain disease which selectively damages the very same brain areas that are affected in AD [6]. Thus, multiple HSV reactivations would lead to a progressive injury in parts of the brain that may favour the clinical expression of AD. The dynamics of the incidence of AD according to IgM status (figure 1) showed an increase more than 7 years after blood sampling. These reactivations do not seem to lead to instantaneous cognitive impairment, but rather may weaken cerebral tissue, leading several years later to AD. This might also explain the absence of association in IgG positive subjects since they were not involved in persistent infection. Other authors have proposed that HSV reactivations could enhance the aggregation of the beta-amyloid protein [24]. Another possibility is a cross-reaction between HSV antibodies and beta-amyloid peptide. Indeed, a significant region of homology between the beta-amyloid protein found in AD and HSV-1 glycoprotein B (gB) has been described [5]. Specifically, gB residues 22–44 (gB22–44) share high similarity to the beta-amyloid carboxyl putative neurotoxic, nucleation and assembly domains. To be IgM positive would then be a marker of the ongoing process of AD pathology.

Another hypothesis is the influence of inflammation due to HSV infection on Alzheimer's disease. In several models of chronic neurodegenerative conditions, a pro-inflammatory response is observed [25] but it is kept under tight control, probably by appropriate anti-inflammatory mediators. The disruption of this anti-inflammatory microglial state has deleterious consequences for the progression of the disease [26], [27]. In a triple-transgenic mouse model of Alzheimer's disease, repeated challenges with lipopolysaccharide were shown to exacerbate central nervous system inflammation and to cause increased tau phosphorylation [28]. As AD pathology begins many years before the dementia stage, recurrent reactivation of HSV might act as a potent stimulus to the brain microglia, increasing the level of cytokines and initiating a positive feedback cycle that gives rise to an increasing accumulation of pathological changes.

If one of both interpretations were true, the prevention of AD by treating or preventing HSV reactivations could be actions to be tested.

An important consequence of our results lies in the fact that subjects with reactivation of HSV are easily detectable through a simple serum analysis which, if repeated over time, could be the basis of primary or secondary prevention strategies towards AD.
